# Consumer Acceptability and Sensory Profile of Sustainable Paper-Based Packaging

**DOI:** 10.3390/foods10050990

**Published:** 2021-05-01

**Authors:** Stella Lignou, Omobolanle O. Oloyede

**Affiliations:** Sensory Science Centre, Department of Food and Nutritional Sciences, University of Reading, Harry Nursten Building, Pepper Lane, Whiteknights, Reading RG6 6DZ, UK; bola.oloyede@reading.ac.uk

**Keywords:** paper-based packaging, sensory attributes, consumer acceptability, biscuit packages, meat packages

## Abstract

Sustainability appears to be increasingly important to consumers. In order for companies to reach their sustainability targets and offer more environmentally friendly solutions to consumers, food producers and retailers have begun to change their packaging to more recyclable, bio-based and biodegradable packaging. This study evaluated the sensory characteristics of paper-based prototype packages developed for two product categories (biscuit and meat packages) using a trained sensory panel. Consumer liking, preference and purchase intent were assessed by 130 participants. For the biscuit packages, no significant differences were observed for the liking of any of the four dimensions assessed (appearance, design, feel or overall liking). However, consumer segmentation identified three relatively homogeneous groups of consumers exhibiting differences in the hedonic reaction to the three packages. For the meat packages, significant differences and preference were observed between the original and paper-based packages. For both categories, the purchase intent was low, indicating that further work needed to be done to improve several quality characteristics (e.g., design, size and strength of the package), which would lead to better consumer acceptability.

## 1. Introduction

Packaging is essential in providing adequate protection to foodstuffs during transport, distribution and storage, thus reducing food loss and waste. Packaging that has a comparatively low environmental impact as assessed by life-cycle assessment models can be considered to be sustainable packaging [1]. From a consumer point of view, a packaging design that evokes explicitly or implicitly the eco-friendliness of the packaging can be considered to be sustainable packaging [2]. Since sustainability appears to be increasingly important to consumers [3,4], market interest in alternative forms to plastic packaging has increased drastically in recent years [5]. In order for companies to reach their sustainability targets and offer more environmentally friendly solutions to consumers, food producers and retailers have started to change their packaging to more recyclable, bio-based and biodegradable packaging. As paper fulfils these requirements and is easily understood by consumers, there is a high market interest for paper-based solutions [5]. 

Paper and cardboard packaging were the most recycled packaging in the UK and Europe in 2018, with recycling rates of 74.4% and 82.9%, respectively [6]. This has motivated companies toward the use of paper-based packaging. In addition, recyclable materials generally give the impression that the packaging is environmentally friendly [7,8,9]. Paper/cardboard is associated with positive emotions and attributes such as trust, biological/natural [10], homely and fresh products [11]. It is generally preferred over plastic because plastic is associated with emotions and attitudes such as unnecessary, expensive or bad for the environment [11].

There is limited research looking at sustainable paper-based packaging while analytically exploring the sensory characteristics of the packaging and consumers’ perceptions. Magnier & Schoormans [12] estimated the effects of visual appearance and verbal sustainability claims on purchase intent and found that consumer responses to the visual appearance and verbal sustainability claims of the package depended on their level of environmental concern. The study showed that consumers with low environmental concerns evaluated conventional-looking packages with a verbal sustainability claim more negatively. In a subsequent study, Magnier et al. [13] investigated the effect of packaging sustainability on consumers’ perceived quality of three product categories and found a more positive perceived quality of a food product when it was packed in a sustainable rather than conventional way. Steenis et al. [14] showed how packaging sustainability influenced consumer perceptions, inferences and attitudes toward packaged products. They demonstrated that consumers often rely on misleading and inaccurate beliefs when judging packaging for sustainability. Most studies acknowledge how the expectations and responses of consumers vary based on the design (shape, orientation, alignment of graphical forms), branding, visual appearance, colour, verbal claims and quality of products [13,15,16,17,18,19]. 

Research has shown that consumers decide what to purchase based on extrinsic product characteristics and appearance [20]. Consumer perception of extrinsic product cues such as packaging material and brand name differs from intrinsic product cues such as aroma, flavour and texture [21]. Packaging and branding as extrinsic product cues have been shown to have an influence on how consumers evaluate food products [22] and can determine consumers’ expectations [23]. Thus, it is important that careful attention is given to the design of a package because of its dual role: attracting consumers’ attention and creating expectations of the sensorial properties of the product [21].

According to a recent systematic review by Ketelsen et al. [24], there were only two studies [25,26] focusing on consumers’ affective liking of environmentally friendly packaging, so research in this area has been quite limited. The study conducted by Koenig-Lewis et al. [25] explored consumers’ emotional and rational evaluations of pro-environmental packages for beverages. Sijtsema et al. [26] investigated consumers’ perceptions of ‘bio-based’ products and found that while participants were unfamiliar with ‘bio-based’ as a concept, they associated the word ‘bio-based’ with both positive and negative sustainability attributes. Therefore, our study (a) evaluated the sensory characteristics of the newly developed paper-based packages for two product categories (biscuit and meat packages), as per Oloyede & Lignou [27] and (b) investigated consumer acceptability, liking and preference of the developed packages and also explored purchase intent.

## 2. Materials and Methods

### 2.1. Materials

Innovative, 3-dimensionally formed paper-based packages were developed for two product categories (biscuits/confectionery and meat/chilled products) using either 3D press forming or deep drawing technology. 

#### 2.1.1. Biscuit Packages

Two paper-based prototypes were developed as an alternative to the traditional polyethylene terephthalate (PET) tray in flow wrap packaging for Strauss Ad Hazot chocolate-coated biscuits. A package of two formed cavities holding three cookies each were individually sealed and easily separable. Sample B1 had a smooth tray surface, whereas sample B2 had an embossed surface. Both versions (B1 and B2) were sealed with a printed lidding film, and trays were cut by twos, with individual trays connected to each other by perforation (Table 1). 

#### 2.1.2. Meat Packages

Three paper-based prototypes were developed to replace an expanded polystyrene (EPS) tray for meat products for Colruyt Group. Sample M1 had an identical shape to the original tray and was formed by deep drawing, sample M2 was press formed with a smooth surface and less-steep side walls, and sample M3 was based on sample M2 with embossing in the bottom area and improved stiffness in the side walls. Samples M1 and M2 had a transparent polymer lidding film with the possibility to see the product, whereas sample M3 had a non-transparent paper-based lidding film (Table 1). 

Life cycle assessment conducted on the paper-based trays with PET coating showed a lower environmental impact compared to plastic crystalline polyethylene terephthalate (CPET) trays and recycled plastic recycled polyethylene terephthalate (rPET) trays [21].

### 2.2. Sensory Evaluation of the Packages

Sensory evaluation was carried out using quantitative descriptive analysis (QDA^TM^) to determine the sensory characteristics of the various prototype packages, and the characteristics were estimated quantitatively. A screened and trained sensory panel (*n* = 12; 11 female and 1 male) was used, and each member had a minimum of 1 years’ experience with expertise in profiling techniques. The panellists received 5 h specific training (1 h per day) over a period of 5 days for each category of packages (biscuits and meat packages) (a total of 10 days for both categories). During the development of the sensory profile, the panellists were asked to describe the appearance and feel of the package and then open the package and describe the interior in order to produce as many descriptive terms as seemed appropriate. The terms were discussed by the panellists as a group, with the help of the panel leader, and this led to a consensus vocabulary of 15 and 16 attributes for biscuit and meat packages, respectively, as outlined in Table 2 and Table 3 in Section 3. The quantitative sensory assessment was carried out in a temperature-controlled room (22 °C) under artificial daylight and in isolated booths, each equipped with an iPad. All panellists scored in duplicate for each sample in separate sessions (30 min each) over two days for each product category. Compusense Cloud Software (Version 21.0.7713.26683, Compusense, Guelph, ON, Canada) was used to acquire the sensory data. In total, 7 samples were evaluated (3 biscuit packages and 4 meat packages in separated sessions). Samples, coded with three-digit random numbers, were provided in a monadic balanced order, with sample sets randomly allocated to panellists within each product category. Panellists were instructed to evaluate the appearance attributes first and then open the package and evaluate the remaining attributes related to the interior of the package. The intensity of each attribute for each sample was recorded on a 100-point unstructured line scale. 

### 2.3. Consumer Evaluation of the Packages

The study was conducted at the Sensory Science Centre at the University of Reading (UK). One hundred and thirty people were recruited across the University of Reading and Berkshire area (male and female, aged 18 years and above, without allergies or intolerances to wheat, gluten and/or dairy). Consumers who took part in the qualitative part of the study [27] were not allowed to sign up. Participants attended a single, 45-min session. Samples were presented to the participants, and after observing the samples, they were asked to rate their liking (appearance, design, feel, overall) on a 9-point hedonic scale (where 1: dislike extremely, 5: neither like nor dislike, 9: like extremely) for all samples. They also indicated the appropriateness of attribute level on a 5-point Just-About-Right (JAR) scale for the following attributes: strength of the package (where 1: much too weak, 3: JAR and 5: much too strong) and naturalness (where 1: not much too natural, 3: JAR and 5: much too natural). Finally, consumers were asked to indicate their preference (ranking: most-preferred to least-preferred package for each category—biscuit or meat packages), purchase intent for the packages (5-point scale, where 1: definitely will not buy, 3: may or may not buy and 5: definitely will buy) and whether they regularly purchased or consumed biscuit or meat (pate) products. Participants were given the opportunity to leave additional comments after evaluating each package if they wanted to. In total, 7 samples were evaluated (3 biscuit packages and 4 meat packages in one session, but with a break between the two product categories). Samples were presented to consumers in a monadic balanced order using Williams design, with sample sets randomly assigned to consumers within each product category. The assessment took place in sensory booths as described in Section 2.2. Consumers were asked to not open the package during assessment. Data was collected using Compusense Cloud Software. The study was conducted in November 2019 and approved by the School of Chemistry, Food and Pharmacy Research Ethics Committee, University of Reading (study number: 51/19). Informed consent was obtained from all participants prior to the study.

### 2.4. Statistical Analysis

SENPAQ version 5.01 (Qi Statistics, Kent, UK) was used to carry out ANOVA of sensory panel data, wherein the main effects (sample and assessor) were tested against the sample by assessor interaction, with sample as a fixed effect and assessor as a random effect. For those attributes exhibiting significant difference in the one-way ANOVA, Fisher’s least significant difference (LSD) test was applied to determine which sample means differed significantly (*p* < 0.05).

XLSTAT 2019.3.2 version (Addinsoft, Paris, France) was used to carry out the following analyses: (i) principal component analysis of the sensory panel data, (ii) one-way ANOVA (and Fisher’s LSD test) for the consumer liking and purchase intent data (iii) analysis of the preference (ranking) data using Friedman’s test; (iv) agglomerative hierarchical clustering (AHC) for overall liking and (v) penalty analysis of the JAR data for strength and naturalness attributes. In more detail, for the AHC, dissimilarity of responses was determined by Euclidean distance, and agglomeration using Ward’s method (set to automatic truncation). For the penalty analysis, the influence of consumer perception of appropriateness of attribute level rating (JAR) on consumer liking was evaluated by calculating the mean drop in liking rating (scale 1–9) compared with mean liking of consumers that rated the attribute as JAR (JAR 3 on a 1–5 scale), determining whether this drop in liking score was significant. 

## 3. Results

### 3.1. Sensory Evaluation of the Packages

#### 3.1.1. Biscuit Packages

Table 2 summarises the mean panel scores of the sensory attributes for the three samples (B0, B1 and B2). All 15 attributes were significantly different between the original package (B0) and the two prototypes (B1, B2). Discrimination, repeatability and consistency were checked for all assessors (Supplementary Data, Table S1). In terms of the appearance attributes, B0 was evaluated as having a more complex design with more amount of text present on the packages because the two prototypes (B1 and B2) had no labels at the back of the package. B0 was quite slippery to hold and the sound of it was very noisy in comparison to B1 and B2. The colour of B0 was dark red and shiny, and the package was very rigid overall. After opening the packages, B0 had many more tears compared to B1 and B2, the inner lid was very shiny and the tray was very rigid, too. Panellists found B1 easier to hold but more slippery to hold compared to the B2 package. Both the B1 and B2 packages did not make any noise and had a matte outer package appearance. B1 was found easier to open compared to B0 and B2. Both B1 and B2 had very shiny inner trays but a less shiny inner lid compared to B0.

**Figure 1:**
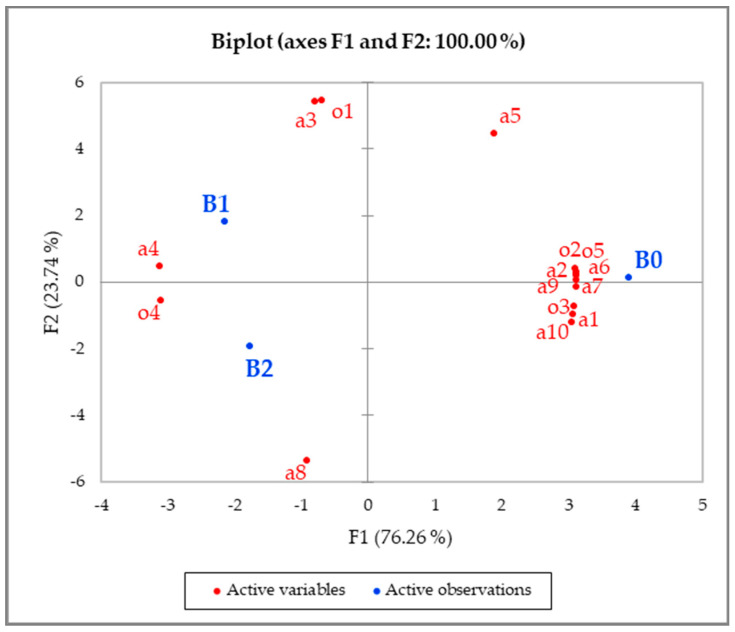
Principal component analysis of biscuit packages (B0, B1 and B2) showing correlations with sensory attributes (codes on plot refer to sensory attribute codes in Table 2).

Principal component analysis was carried out on the correlation matrix of all samples and all attributes in order to graphically visualise the differences between the samples. The first two principal components accounted for all the variation in the data (Figure 1). The first axis (76.26%) mainly separated B0 from the two prototypes, whereas the second axis separated the two prototypes—B1 and B2 (23.74%). The majority of the attributes were positively correlated with the first axis and thus associated with B0. Important attributes included the complexity of the design, the amount of text, the brightness of the colour of the package, the noise of the package and the rigidity of the tray before and after opening the package. On the other hand, the two prototypes had a shinier inner tray and sharp edges. The B2 package had a rougher bottom surface, whereas B1 was easier to open. 

#### 3.1.2. Meat Packages

Table 3 summarises the mean panel scores of the sensory attributes for the four meat packages (M0–M4). All 16 attributes were significantly different among the original package and the three prototypes. It could be observed that the M0 was quite deep with a very shiny and rigid tray. The lid was quite tightly sealed on the top of the package, and overall, the package was quite stable when placed on a table. After opening the packages, M0 was quite easy to open but tears developed on the lid. The tray was still quite rigid even after removing the lid, and the inner tray was found to be less shiny compared to the other packages (M1–M3). The three paper-based prototypes (M1–M3) were less deep compared to the original package, had a cream colour and were not shiny. M3 was quite rigid before opening the package and exhibited similar scores to M0 in terms of the tightness of the lid and stability when placed on a table. 

**Figure 2:**
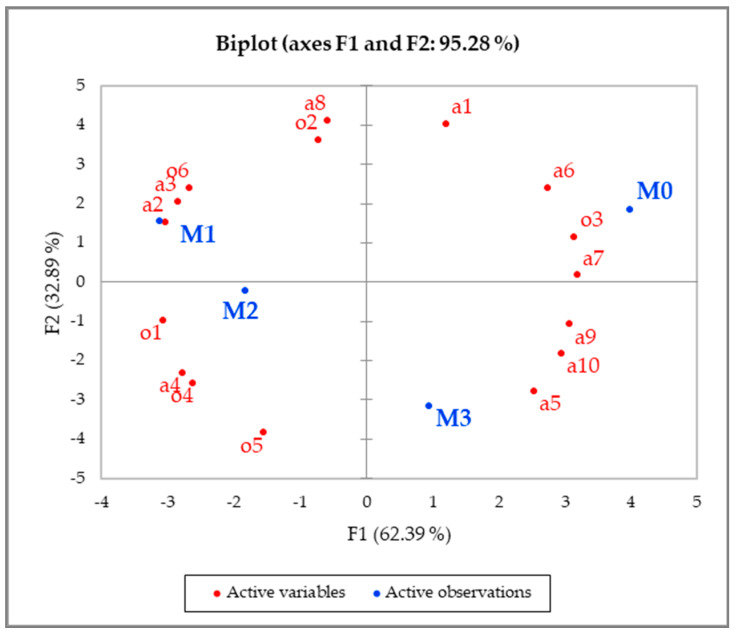
Principal component analysis of meat packages (M0, M1, M2, and M3) showing correlations with sensory attributes (codes on plot refer to sensory attributes codes in Table 3).

Similar to biscuit products, principal component analysis was carried out in order to graphically visualise the differences between the meat packages. The first two principal components accounted for 96.3% of the variation in the data (Figure 2). The first axis mainly separated M1 and M2 from M0, whereas the second axis separated M3 from the rest of the packages. Attributes positively correlated with the first axis, and thus associated with the M0 package, were the rigidity of the tray before and after opening the package, the shininess of the outer tray, the depth of the package, the tightness of the lid and sitting of the tray on the table. On the other hand, attributes negatively correlated with the first axis and thus associated with the M1 and M2 packages were the difficulty of separating the barrier and the difficulty of opening the package as well as the ability to hold the package and the level of slipperiness when holding the package. Transparency of the lid attribute positively correlated with the second axis and was negatively correlated with M4 packages because the lid was not transparent at all. 

### 3.2. Consumer Evaluation of the Products

Table 4 summarises the demographic data for the consumers. One hundred and thirty consumers evaluated the samples. A higher proportion of the consumers were female (72.3%), and the mean and median ages were 32.8 and 29, respectively. More than one-third of the consumers were working (36.9%), and 58.5% were students. In total, 47.7% of the consumers that took part were people connected with the food, nutrition or sensory sector. The largest ethnic group to participate were White British (40%). The majority of the participants consumed or purchased biscuits sometimes or frequently (78.5%), whereas for the meat packaging, and particularly for pate (as this was the meat product inside the package), only 33.8% of the participants consumed or purchased it sometimes or frequently.

#### 3.2.1. Biscuit Products

The mean liking scores of the packages are presented in Table 5. The results show that there were no significant differences in the appearance, design, feel and overall liking for all the samples tested, with all results ranging between like slightly and like moderately. While consumers did not like any of the packages very much, the results showed that both original and new packages were liked at a similar level, which can be seen as a positive for the new paper-based packages, to some extent.

In order to identify relatively homogeneous groups of consumers, agglomerative hierarchical cluster analysis was conducted, and three clusters of consumers were identified (Table 6). Consumers in cluster 1 (40.8%) liked slightly the original package of the biscuits and less the paper-based packages (B1 and B2). Cluster 2 (50%), the largest cluster, liked all three samples, whereas cluster 3 (9.2%), did not like B0 but liked moderately the paper-based packages. 

Consumers were also asked to rank the samples in order of overall liking with 1-most liked and 3-least liked (Table 5). The results from the Friedman’s test showed that there was no significant difference in preference ranking of overall liking of all the three packages, a result that it is in agreement with the non-significant result obtained for overall liking.

Penalty analysis was used to relate JAR data to liking scores and explain drivers of liking in relation to strength and naturalness, and the results are presented in Table 7. There was no significant difference in the JAR strength of the packages, and all three packages were perceived very close to Just-About-Right (JAR = 3). However, a significant difference was observed for the JAR naturalness, with packages B1 and B2 considered closer to Just-About-Right compared to B0. 

When the attributes are not at the optimum level for a consumer this may have an effect on the overall liking. The penalty analysis showed that for samples B1 and B2 there was a negative impact on the overall liking when the strength of the package was considered too low. Similarly, for naturalness, there was a significant drop in the liking of all the packages when the naturalness of the package was considered to be ‘too little’ by the consumers with B1 considered to be the least natural of all the packages. 

Finally, consumers were asked about their purchase intent of these packages (5-point scale: 1-definitely will not buy, 2-probably will not buy, 3-might or might not buy, 4-probably will buy and 5-definitely will buy). The mean scores of the purchase intent for all three packages ranged between 3.3-3.6 (Table 5), and a significant difference was observed (*p* = 0.039), with consumers more likely to buy B0 than B2. There were no significant differences between B1 and B2 (*p* = 0.636) or B0 and B1 (*p* = 0.110). Additional comments on the packages provided by the participants were both positive and negative. Some examples of those comments are shown in Table 8.

#### 3.2.2. Meat Packages

The mean liking scores of the meat packages are presented in Table 5. As can be observed, there were significant differences in all four liking dimensions. The appearance, design and overall liking of M0 were significantly higher than all the paper-based packages. No significant differences were observed between M2 and M3 for appearance, design or overall liking. In terms of the liking of the feel, the feel of M0 was significantly more liked than M1 and M3, but not M2.

Similar to the biscuits, AHC results are presented in Table 9 for the meat packages. Consumers in cluster 1 (27.7%) slightly liked the original package of the meat and disliked moderately to slightly the transparent film paper-based packages (M1 and M2), whereas they disliked very much the non-transparent paper-based package (M3). Cluster 2 (53.8%), the largest cluster, liked slightly M0 and M2, followed by M3 and M1. Finally, cluster 3 (18.5%) disliked very much the paper-based packages with transparent film (M1 and M2) and neither liked nor disliked the other two packages. 

When consumers were asked to rank their preference in terms of overall liking, significant differences (*p* < 0.0001) were observed (Table 5). M0 significantly differed from all the other packages and was the most preferred. On the other hand, M1 and M3 did not differ significantly and were the least preferred of all. This result was again in agreement with the overall liking results discussed earlier.

Significant differences in Just-About-Right strength and naturalness attributes were observed for the four packages (Table 7). In terms of strength, M0 was perceived just above Just-About-Right (JAR = 3), whereas for the other three samples, the strength of the packages were considered ‘not too strong’. For the naturalness attributes, the M2 sample was close to Just-About-Right, whereas the naturalness of M0 was considered ‘not too natural’. The penalty analysis showed that for samples M1 to M3, there was a negative impact on the overall liking when the strength of the package was considered too low. Similarly, for naturalness, there was a significant drop in the liking of all the packages when the naturalness of the package was considered to be ‘too little’ by the consumers, with M0 considered to be the least natural of all the packages. 

Finally, in terms of purchase intent, the mean scores for all the paper-based packages ranged between 2.4–2.8 (Table 5), which implied that consumers did not generally like the design of those packages. On the other hand, the purchase intent for samples M0 was at 3.3, between ‘might or might not buy’ and ‘probably will buy’. Similar to the biscuit packages, participants’ comments on the packages were both positive and negative, and examples of those comments are shown in Table 8.

## 4. Discussion

The present study aimed to (1) explore the sensory characteristics of the new paper-based packages developed during the study for two product categories (biscuits and meat) in comparison to the original packages, as assessed by a trained panel and (2) evaluate consumers’ liking and perceptions of the said packages. The findings from this study build on and contribute to existing knowledge on consumer opinions and reactions to paper-based packaging material [27].

For the biscuit packages, no significant differences were observed for the liking of any of the four dimensions (appearance, design, feel or overall liking); however, consumer segmentation identified three relatively homogeneous groups of consumers exhibiting differences in hedonic reaction for the three packages. Even though no significant preference was observed (*p* = 0.299), consumers in each cluster varied in their responses. Consumers in cluster 2 (50%) “liked moderately” all three packages but seemed to “like significantly” more the new paper-based packages. Similarly, the paper-based packages were liked more by the consumers in cluster 3 (9.2%) who disliked the original package (B0). In a study conducted by Fernqvist et al. [11] exploring consumers’ views on different aspects of fruit and vegetable packaging, the authors found that the design of the package was interpreted differently among participants. While some participants had a positive perception about the package, others had a negative opinion. Consumers were given the opportunity to add comments for the various packages, and it was clear that they appreciated the innovative packages of B1 and B2, and they loved the duo-pack design that meant a separation of the packages and that the consumption of a smaller portion was possible while keeping the other portion ‘*fresh, crisp and for longer*’. As expected, the paper-based packages had a more natural and sustainable feel when compared to the B0 package, and this was apparent from the Just-About-Right ratings and consumers’ comments: ‘*it feels very natural*’, ‘*it looks sustainable*’, ‘*the packaging seems more natural and biodegradable*’. The results also demonstrated that there was a significant drop in the overall liking of the package when the naturalness was considered to be ‘too little’. Prior research has shown that sustainability perceptions can be closely related to other benefits such as naturalness [13], which is a positive characteristic of sustainable packaging. 

Focusing on the characteristics of the paper-based packages, it seemed that even though consumers liked the smoothness of the B1 bottom surface and its ‘*sustainable look and nice feel*’, they thought the tray was not too rigid and was a bit fragile. This was confirmed from the sensory evaluation results, wherein trained panellists scored B1 significantly lower (43.7) for rigidity before opening the package compared to the original package (69.2), and also from the significantly lower score in terms of the JAR strength attribute. The perception of the rigidity of the package was further reduced to 30.8 after opening the package and removing the lid. On the other hand, the B2 tray had an embossed bottom surface, which consumers felt was ‘easy to hold’ and was seen as a positive characteristic. This was also confirmed by the trained panel wherein the level of perceived slipperiness was significantly lower (21.3) for B2 compared to the B0 and B1 samples. The perception of fragility may have had an effect on consumers’ acceptability of the B1 package, as it may have been seen as a quality issue of the package that could affect its ability to protect its content. 

There was also a cluster of consumers (cluster 1—40.8%) that significantly liked the original package compared to the new packages. These were consumers who preferred to go with what they were familiar with and were less keen to try new propositions. Some of the consumers in this group had comments such as ‘*love the compact design*’, ‘*seems like the standard design so keen to buy*’, ‘*I am familiar with this packaging*’, ‘*it immediately reminds me of biscuits, which I like*’. Most consumers tend to be creatures of habit and unwilling to try new things, as found by Oloyede & Lignou [27]. In addition, consumers have an expectation of what the package design should be like and would generally be averse to trying designs that do not match the picture they have in their minds. Zhang et al. [19] reported that the design style or colour of the package of UHT milk was shown to have an influence on consumer attraction. The authors suggested that if consumers are more attracted to the design style or colour, their willingness to purchase will be higher. Ares and Deliza [17] showed that package shape and colour could have an impact on consumers’ expected liking scores and their sensory expectations in a product category such as desserts, and similar results were demonstrated with this study. The relevance of package characteristics, in this case the shape of a standard biscuit package, had an effect on consumers’ perception and acceptance and also on purchase intent. Consumers were more likely to buy the original package as earlier discussed. 

Regarding the meat packages, significant differences were observed for appearance, design, feel and overall liking with subsequent significant preference of certain packages over others (*p* < 0.0001). In general, consumers liked the original package (M0) more than the paper-based packages (M1–M3); however, similar to the biscuits, consumer segmentation identified three clusters of consumers with varying overall liking for the four packages, which was clear from the comments they added. Consumers in the largest cluster (cluster 2—53.8%) equally liked M0 and M2 when compared to M1 and M3. Consumers felt that the polystyrene of M0 ‘*evokes hygiene—associated with meat*’. They liked the feel of the packaging, how sturdy and deep it was and the fact that the lid on top was not in direct contact with the meat. This result agrees with the findings of Oloyede & Lignou [27], wherein focus group participants were worried about contamination due to the top lid touching the meat. This was also confirmed by the trained panel, who scored significantly higher the depth of this package (72.6) and the rigidity before and after opening the package (94.6 and 93.2, respectively) compared to the other three packages. The overall liking in the other two clusters was mainly driven by whether the top lid was transparent or not. For example, consumers in cluster 1 (27.7%) disliked very much M3, equally disliked M2 and M3 and liked slightly M0, whereas consumers in cluster 3 (18.5%) equally disliked very much M1 and M2 and neither liked nor disliked M0 and M3. 

Interestingly, no matter the cluster, the M1 and M2 packages had very similar characteristics in general, which was confirmed from the sensory evaluation. Both samples had a smooth bottom surface that resulted in significantly higher perceived ability to hold, level of slipperiness and very low rigidity before and after opening the packages compared to M0 and M4. Some consumers liked this feel and stated that it ‘*looked very neat*’. The only differences observed between the two packages was the difficulty of opening the package and the difficulty in separating the inner barrier, with both receiving a higher rating for the M1 package. Observing the results for the M3 package, it seemed that on one hand, consumers preferred the embossed packaging tray over the non-embossed due to the touch and feel of the paper, the sturdiness and the fact that it made the packaging look more attractive (5.6 hedonic liking for cluster 2 and 5.0 for cluster 3); however, it was clear that for certain consumers, the lidding material and its transparency was crucial (2.1 hedonic liking for cluster 1), as consumers in general prefer to see the content of the packaging [28], and especially when the product is meat. Transparent packaging has been shown to increase willingness to purchase, expected freshness and expected quality in different food categories (cereal, boxed chocolates, dried pasta and fresh fish) [29]. Consumers mentioned that there was a minimalistic feel associated with M3, and they liked the fact that it was all paper and no plastic; however, they worried that the paper package might absorb moisture or meat blood/liquid with time. These findings agree with the study by Magnier and Crie [2], who found that eco-friendly packages, because of their simplicity, minimalism and lack of colours, are often perceived as less appealing.

The results show that the positive and negative perceptions regarding the paper-based packages had an effect on the overall liking of the products, which in turn affected the purchase intent. The mean scores of the purchase intent for all three paper-based packages ranged between 2.4–2.8, which is between ‘probably will not buy’ and ‘might or might not buy’, implying that consumers did not generally like the design of these packages.

There are a couple of limitations to this study. Given the limited duration of the project, there was insufficient time to completely develop the packages and include all the relevant information regarding the labelling of the products. The biscuit packages, other than the red cover which had the same graphics as the original package, had no further information on the nutritional profile of the content or any information regarding the recyclability of the actual package. For the meat packages, the situation was even more complicated because there was no information at all about the product. Previous research has shown that consumers’ responses to either visual or verbal responses can vary depending on cognitive resources [30]; however, in our case, no cues were provided to the consumers. Future research with packages having all the relevant information needed by the consumers printed on the package would allow for better comparisons, not only of the design and feel of the package/material but also the appearance and the messages to be delivered to the consumers. 

## 5. Conclusions

The results from the sensory and consumer evaluation of the new paper-based packages clearly demonstrated that these packages were a good example of how paper can be used as an alternative to plastic or foil for the development of packages in product categories such as confectionery or chilled products. In summary, consumers liked the sustainable nature of the paper-based packages; however, they found the trays (particularly for the meat packages) to be flimsy and not strong enough. For the biscuits, they liked the innovative design of the double pack but also loved the compact design of the original package, as this was more familiar to them and looked like a standard pack of biscuits. The results showed that while consumers were open to sustainable propositions, other quality characteristics were key aspects that must be addressed if sustainable packaging is to become a viable option. From an industry point of view, considerations have to be based not only on the sustainable nature of the packaging material but also on the design and size of the packaging. This is because design and size of package are more important factors influencing consumer choice than the sustainable character of the packaging material. Thus, further work needs to be done to improve several quality characteristics (e.g., design and size of package), which would lead to better consumer acceptability. 

## Figures and Tables

**Table 1:** Biscuit and meat packages.

Samples			
*Biscuit packages*			
B0: preformed polymer multicavity tray, polymer flow pack (horizontal) 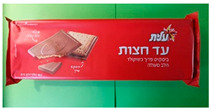	B1: form-fill-seal paper-based tray with paper-based lidding film 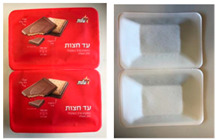		B2: form-sill-seal paper-based tray with paper-based lidding film 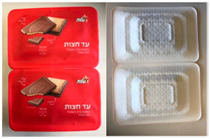
*Meat packages*			
M0: preformed polymer tray with polymer lidding film 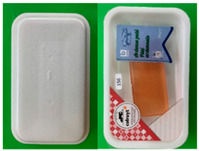	M1: preformed paper-based tray with polymer lidding film 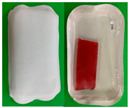	M2: form-sill-seal paper-based tray with polymer lidding film 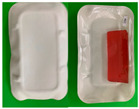	M3: form-fill-seal paper-based tray with paper-based lidding film 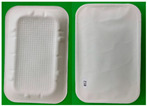

**Table 2:** Mean panel scores for sensory attributes of the three biscuit packages.

Code	Attributes [Anchors 0–100)]	Scores ^1^			LSD ^2^	*p*-Value ^3^
		B0	B1	B2		
	*Appearance*					
a1	Complexity of design (top and bottom) [simple to complex]	65.5a	28.3b	37.1b	8.9	<0.0001
a2	Amount of text [low to high]	72.5a	25.1b	25.4b	6.9	<0.0001
a3	Ease of holding [easy to difficult]	25.6ab	34.9a	21.2b	10.1	0.0313
a4	Sharp edges [not to very]	2.0b	35.6a	30.6a	13.1	0.0001
a5	Level of slipperiness [not to very]	42.7a	38.7a	21.3b	12.0	0.0032
a6	Noise of package [quiet to noisy]	68.3a	5.0b	6.2b	6.5	<0.0001
a7	Brightness of colour [light to dark]	63.8a	29.1b	30.7b	11.7	<0.0001
a8	Roughness of bottom surface [smooth to rough]	17.0b	10.2b	47.2a	15.8	0.0002
a9	Shininess of outer package [matt to shiny]	48.7a	10.1b	13.3b	9.2	<0.0001
a10	Rigidity before opening the package [flimsy to rigid]	69.2a	43.7b	50.8b	9.4	<0.0001
	*After opening the package*					
o1	Difficulty of opening [easy to difficult]	31.0b	47.3a	22.1b	12.7	0.0018
o2	Tearing [none to lots]	44.3a	0.0b	0.0b	10.5	<0.0001
o3	Rigidity of the tray after opening the package [not to very]	73.3a	30.8b	39.1b	10.9	<0.0001
o4	Shininess of the inner tray [matt to shiny]	52.8b	72.8a	73.8a	11.7	0.0017
o5	Shininess of the inner lid [ matt to shiny]	79.1a	69.9b	69.7b	6.5	0.0097

^1^ Means not labelled with the same letters are significantly different (*p* < 0.05); means are from two replicate samples, measured on an unstructured line scale (0–100). ^2^ Fisher’s least significance difference (LSD) at *p* = 0.05. ^3^ Probability, obtained from ANOVA, that there is a difference between the means.

**Table 3:** Mean panel scores for sensory attributes of the four meat packages.

Code	Attributes [Anchors 0–100)]	Scores ^1^				LSD ^2^	*p*-Value ^3^
		M0	M1	M2	M3		
	*Appearance*						
a1	Depth of tray [not to very]	72.6a	54.5b	28.1c	18.8c	9.4	<0.0001
a2	Ability to hold [easy to difficult]	16.4b	42.7a	37.0a	17.8b	11.3	<0.0001
a3	Level of slipperiness [not to very]	24.1b	56.2a	48.9a	19.8b	16.1	0.0001
a4	Colour of the tray [white to cream]	1.0b	84.7a	84.6a	82.4a	13.8	<0.0001
a5	Roughness of bottom surface [smooth to rough]	47.4b	6.7d	23.2c	63.7a	12.3	<0.0001
a6	Shininess of outer package [matt to shiny]	51.5a	5.4b	2.4b	5.0b	10.1	<0.0001
a7	Rigidity before opening the package [flimsy to rigid]	94.6a	31.3c	30.2c	66.0b	9.7	<0.0001
a8	Transparency of lid [not to very]	99.7a	97.0a	98.3a	0.0b	2.9	<0.0001
a9	Tightness of lid [not to very]	97.2a	50.2b	51.9b	90.7a	13.4	<0.0001
a10	Sitting of tray on the table [not stable to stable]	96.7a	26.8c	58.0b	95.3a	12.1	<0.0001
	*After opening the package*						
o1	Difficulty of opening [easy to difficult]	14.4c	93.4a	68.2b	65.2b	17.3	<0.0001
o2	Tearing [none to lots]	30.5ab	43.8a	15.2bc	12.5c	16.6	0.0018
o3	Rigidity of the tray after opening the package [not to very]	93.2a	19.5c	23.8c	43.7b	10.8	<0.0001
o4	Thickness of the lid [thin to thick]	18.5b	59.0a	54.3a	60.5a	9.7	<0.0001
o5	Shininess of the inner tray [matt to shiny]	51.4c	71.5b	72.4b	87.2a	9.4	<0.0001
o6	Difficulty of separating barrier [easy to difficult]	36.8c	79.7a	53.8b	31.1c	16.2	<0.0001

^1^ Means not labelled with the same letters are significantly different (*p* < 0.05); means are from two replicate samples, measured on an unstructured line scale (0–100). ^2^ Fisher’s least significance difference (LSD) at *p* = 0.05. ^3^ Probability, obtained from ANOVA, that there is a difference between the means.

**Table 4:** Consumer demographics and characteristics of consumer panel.

Consumers	Number	Percentage (%)
Total number of volunteers	130	
*Age*		
mean	32.8	
median	29	
min	18	
max	66	
*Gender*		
male	36	27.7
female	94	72.3
*Working status*		
working	48	36.9
unemployed	0	
student	76	58.5
other	6	4.6
working in food/nutrition/sensory sector	62	47.7
*Ethnic group*		
White British	52	40.0
White other	35	26.9
Mixed	1	0.8
Indian, Pakistani, Bangladeshi	8	6.2
Chinese	11	8.4
African, Caribbean	3	2.3
Arab	6	4.6
Other	13	10.0
Not declared	1	0.8
*Frequency of biscuit consumption/purchase*		
Frequently (approx. once per week)	42	32.3
Sometimes (approx. once per month)	60	46.2
Rarely (less than once per month)	25	19.2
Never	3	2.3
*Frequency of pate consumption/purchase*		
Frequently (approx. once per week)	12	9.2
Sometimes (approx. once per month)	32	24.6
Rarely (less than once per month)	51	39.2
Never	35	26.9

**Table 5:** Liking scores, preference ranking and purchase intent for biscuit and meat packages.

Code	Liking ^1^				Ranking ^2^	Purchase Intent ^3^
	Appearance	Design	Feel	Overall		
*Biscuit packages*						
B0	6.7	6.5	6.5	6.6	1.9	3.62b
B1	6.4	6.3	6.6	6.5	2.1	3.41ab
B2	6.3	6.4	6.4	6.4	2.0	3.35a
*p*-value	0.099	0.558	0.657	0.540	0.299	0.096
*Meat packages*						
M0	6.3a	6.4a	5.6a	6.1a	1.7a	3.33a
M1	4.8b	4.7b	5.0b	4.9b	3.0c	2.79b
M2	4.2c	4.5c	5.4ab	4.5bc	2.2b	2.35c
M3	4.0c	4.0c	4.4c	4.2c	3.0c	2.35c
*p*-value	<0.0001	<0.0001	<0.0001	<0.0001	<0.0001	<0.0001

^1^ Means not labelled with the same letters are significantly different (*p* < 0.05); means are from 130 consumers on a 9-point hedonic scale (from dislike extremely to like extremely). ^2^ Mean rank (1: most preferred to 3: least preferred). ^3^ Measured on a 5-point scale (1: definitely will not buy to 5: definitely will buy).

**Table 6:** Overall liking of the biscuit packages for the clusters of consumers obtained from agglomerative hierarchical clustering.

Cluster/Percentage of Consumers	Samples ^1^			*p*-Value
	B0	B1	B2	
1 (40.8%)	6.3a	5.1b	4.7b	<0.0001
2 (50.0%)	7.4b	7.7a	7.7a	0.057
3 (9.2%)	4.2b	7.1a	7.3a	<0.0001
Overall liking	6.6	6.5	6.4	0.540

^1^ Means not labelled with the same letters are significantly different (*p* < 0.05); means are from 53 consumers for cluster 1, 65 consumers for cluster 2 and 12 consumers for cluster 3, respectively. The mean for overall liking is from 130 consumers.

**Table 7:** Mean Just-About-Right ratings and influence on overall liking ratings.

Packages	Overall	Significance of Sample (*p*-Value)	Penalty Analysis			
			Too Little		Too Much	
			Mean Drop	Frequency (%)	Mean Drop	Frequency (%)
*Biscuit Packages*						
JAR Strength						
B0	3.03b	0.107	0.57	10.0	0.11	11.5
B1	2.87a		1.49 *	25.4	0.64	13.9
B2	2.91ab		2.08 *	22.3	1.03	14.6
JAR Naturalness						
B0	2.18a	<0.0001	1.05 *	63.9	−0.16	3.9
B1	2.82b		1.25 *	22.3	−0.24	9.2
B2	2.77b		2.49 *	24.6	0.69	6.9
*Meat Packages*						
JAR Strength						
M0	3.14a	<0.0001	2.17	7.7	0.40 *	20.8
M1	2.47b		1.61 *	60.8	1.42	6.9
M2	2.37b		2.03 *	49.2	1.41	5.4
M3	2.31b		1.53 *	53.1	1.14	3.1
JAR Naturalness						
M0	2.12c	<0.0001	1.84 *	64.9	−0.39	4.6
M1	2.42b		2.19 *	54.6	1.1	7.7
M2	2.90a		1.99 *	53.9	0.82	6.9
M3	2.35b		2.04 *	24.6	0.37	17.7

* Represents a significant difference (*p* < 0.05) within a sample in overall liking compared with mean liking rating when the sample was considered Just-About-Right. Frequency (%) is the % of participants within each group.

**Table 8:** Examples of participants’ comments (one positive and one negative comment) relating to the various packages.

Sample	Comments and Participants Details
*Biscuit packages*	
B0	*This package looks so common (IP60, female, aged 24). I would avoid this one if I was trying to reduce my waste and carbon footprint, unless it was advertised as biodegradable (IP70, female, aged 30).*
B1	*I think the paper packaging makes the product seem of a higher value than plastic (IP63, female, aged 22). Makes me think the quantity in the package might not be big enough (IP72, male, aged 29).*
B2	*Nice paper packaging and texture (IP78, male aged 36). Packaging seems a little bit too thick and heavy duty for a simple biscuit packaging (IP69, male, aged 18).*
*Meat packages*	
M0	*Film cover seems strong (IP21, female, aged 52). Looks like standard package, I just hate polystyrene (IP36, female, aged 46).*
M1	*Does look sufficiently sealed and would be prepared to buy if it was ‘the norm’ or environmentally friendly (IP38, male, aged 58). Looks cheap (IP22, female, aged 52).*
M2	*Package seems natural. No harmful toxic effects (IP23, female, aged 34). Not very eye catching (IP21, female, aged 52).*
M3	*Liked the natural feel of the paper tray (IP8, female, aged 21). Not a visible package (IP26, female, aged 24).*

**Table 9:** Overall liking of the meat packages for the clusters of consumers obtained from agglomerative hierarchical clustering.

Cluster/Percentage of Consumers	Samples ^1^				*p*-Value
	M0	M1	M2	M3	
1 (27.7%)	6.7a	3.8b	4.1b	2.1c	<0.0001
2 (53.8%)	6.3a	5.2c	6.0ab	5.6bc	0.0005
3 (18.5%)	4.8a	2.0b	2.8b	5.0a	<0.0001
Overall liking	6.1a	4.9b	4.5bc	4.2c	<0.0001

^1^ Means not labelled with the same letters are significantly different (*p* < 0.05); means are from 36 consumers for cluster 1, 70 consumers for cluster 2 and 24 consumers for cluster 3, respectively. The mean for overall liking is from 130 consumers.

## Data Availability

The data presented in this study is available on request from the corresponding author.

## Supplementary Materials

The following are available online at https://www.mdpi.com/article/10.3390/foods10050990/s1, Table S1: Summary table of assessor performance for biscuit and meat packages.
