# Supplementary material for: Consumer Acceptability and Sensory Profile of Sustainable Paper-Based Packaging
Source: Foods. 2021 May 1;10(5):990. doi: 10.3390/foods10050990 (PMC8147313; doi:10.3390/foods10050990)
Supplement: Supplementary file 1 [file foods-10-00990-s001.zip › foods-1187610-supplementary.pdf]

Table S1. Summary table of assessor performance for biscuit and meat packages.

| **Assessors' performance** | **1** | **2** | **3** | **4** | **5** | **6** | **7** | **8** | **9** | **10** | **11** |
|---|---|---|---|---|---|---|---|---|---|---|---|
| *Biscuit packages* | | | | | | | | | | | |
| Non-discrimination of attributes | 16 | 18 | 18 | 18 | 18 | 17 | 18 | 17 | 17 | 14 | 16 |
| Non-repeatability of attributes | 3 | 4 | 2 | 2 | 1 | 2 | 1 | 2 | 0 | 0 | 0 |
| Attributes causing interaction | 8 | 7 | 4 | 8 | 5 | 6 | 10 | 4 | 3 | 7 | 9 |
| *Meat packages* | | | | | | | | | | | |
| Non-discrimination of attributes | 5 | 3 | 5 | 9 | 3 | 7 | 8 | 4 | 9 | 3 | 3 |
| Non-repeatability of attributes | 1 | 1 | 0 | 3 | 0 | 1 | 6 | 0 | 2 | 0 | 3 |
| Attributes causing interaction | 6 | 5 | 8 | 5 | 2 | 5 | 9 | 4 | 6 | 4 | 6 |
